# Ascorbic Acid in Colon Cancer: From the Basic to the Clinical Applications

**DOI:** 10.3390/ijms19092752

**Published:** 2018-09-13

**Authors:** Ibrahim El Halabi, Rachelle Bejjany, Rihab Nasr, Deborah Mukherji, Sally Temraz, Farah J. Nassar, Haidar El Darsa, Ali Shamseddine

**Affiliations:** 1 Department of Internal Medicine—Division of Hematology-Oncology, American University of Beirut Medical Center, Riad El Solh, Beirut 1107 2020, Lebanon; ije03@mail.aub.edu (I.E.H.); rb62@aub.edu.lb (R.B.); dm25@aub.edu.lb (D.M.); st29@aub.edu.lb (S.T.); fjn00@mail.aub.edu (F.J.N.); he53@aub.edu.lb (H.E.D.); 2 Department of Anatomy, Cell Biology and Physiology, American University of Beirut, Beirut 1107 2020, Lebanon; rn03@aub.edu.lb

**Keywords:** CRC, vitamin C, AA, cell lines

## Abstract

Given the safety and potential benefits of intravenous ascorbic acid (AA) administration in cancer patients, there is merit in further exploring this therapeutic concept. In this review, we discuss the potential benefits of intravenous AA administration on colorectal cancer and we specifically focus on its effect on glycolysis in mutant and wild type *RAS*. We perform a PubMed and Ovid MEDLINE search using ascorbic acid, intravenous vitamin C, *KRAS* mutation, *BRAF* mutation and colorectal cancer (CRC) as keywords. At the cellular level, colorectal cancer cells undergo a metabolic shift called the Warburg effect to allow for more glucose absorption and utilization of glycolysis. This shift also allows AA to enter which leads to a disruption in the Warburg effect and a shutdown of the downstream *KRAS* pathway in mutated *KRAS* colon cancer cells. At the clinical level, AA is associated with tumour regression in advanced disease and improved tolerability and side effects of standard therapy. Based on these findings, we conclude that further clinical trials are needed on a larger scale to examine the therapeutic benefits of AA in colon cancer.

## 1. Background

Colorectal cancer (CRC) is one of the three most common cancer types in men and the second in women; on a yearly average, one million people are diagnosed with colorectal cancer worldwide [1]. The mortality rate varies significantly by disease stage; with a 5-year survival rate in more than 90% of stage I patients, ranging from 10–20% in metastatic patients [2]. CRC is a heterogeneous disease in which about 40% of cases carry a *KRAS* mutation and 10% are characterized by a *BRAF* mutation [3]. Mutant *KRAS* is associated with poor outcomes, poor overall survival [4] and resistance to anti-epidermal growth factor receptor (EGFR) therapy compared to wild type *KRAS* patients [5,6]. Several decades ago, vitamin C (also known as ascorbic acid; AA) was introduced as a therapy for cancer. It has shown potential benefits on the tumour response, improved survival and provided better quality of life [7].

The purpose of this review is to explore the therapeutic concept of intravenous administration of AA in patients with CRC. We go over the potential benefits of its use, emphasizing on its effect on glycolysis in tumours harbouring mutant and wild type *RAS*.

## 2. Methods

We searched Ovid MEDLINE and PubMed using ascorbic acid, intravenous vitamin C, *KRAS* mutation, *BRAF* mutation and colorectal cancer as keywords. We also searched bibliographies manually.

## 3. Results

### 3.1. Glycolysis in Colorectal Cancer

While normal cells rely on mitochondrial oxidative phosphorylation to generate ATP, cancer cells employ an alternative method to produce energy, termed the Warburg effect (aerobic glycolysis) [8]. This effect refers to a metabolic shift whereby glucose is absorbed at a faster rate, partly due to the overexpression of glucose transporters and lactic acid is produced rather than pyruvate due to the overexpression of specific glycolytic enzymes [9]. This basically leads to the utilization of glycolysis rather than oxidative phosphorylation. This metabolic reprogramming in cancer cells was recently introduced as a new hallmark of cancer [10].

Studies on colon cancer cells have revealed that Wingless-related integration site (Wnt) signalling, which is essential for tissue development, if altered, plays a key role in the Warburg effect and angiogenesis as well. Pate et al. conducted several experiments to investigate how blocking Wnt signalling affects colon cancer cells. These studies revealed a decrease in lactate production, an increase in ATP through oxidative phosphorylation and a decrease in glucose absorption, thereby inhibiting the Warburg metabolic shift. Furthermore, it was demonstrated that pyruvate dehydrogenase kinase isozyme 1 (PDK1) is a key downstream product of Wnt signalling. Overexpression of PDK1 reverses the effects of Wnt blockage and allows the Warburg effect to continue. Finally, blocking Wnt signalling was also found to reduce tumour vessel density, hence suggesting a possible role for Wnt signalling in angiogenesis [11].

Cytokines such as interleukin-17 (IL-17), tumour necrosis factor alpha (TNF-α) and IL-6 have also been implicated in promoting aerobic glycolysis [12]. Liu et al. examined the role of IL-22 in colon cancer cells. They determined a link between inflammation and the Warburg effect via the pro-inflammatory cytokine IL-22 which amplified aerobic glycolysis by targeting the hexokinase-2 (HK-2) gene and pathway. IL-22 was also found to upregulate *C*-*MYC* the main transcription factor responsible for the regulation of glycolytic genes, which, in turn, was found to be responsible for IL-22 mediated HK-2 upregulation. This effect was not seen in hypoxia-inducible transcription (HIF)-1 alpha, a transcription factor that is also responsible for glycolytic gene regulation. Furthermore, IL-22 induced signal transducer and activator of transcription (STAT)-3 activation by phosphorylation which enhanced aerobic glycolysis as well [8].

In summary, colon cancer shifts the method of obtaining energy to the Warburg effect, which relies primarily on glycolysis and this is augmented by several signalling pathways, glycolytic enzymes and pro-inflammatory cytokines.

### 3.2. Pharmacokinetics of Ascorbic Acid

The urinary excretion of ascorbate was shown to be reduced in patients with cancer when compared to healthy persons. After a continued daily oral intake of 400 mg of AA, Spellberg and Keeton showed that cancer patients excrete 34 to 48% of intake, whereas healthy individuals excrete 56 to 80%. Similar findings were found in a study conducted by Bodansky, in which the daily urinary excretion of AA in cancer patients ranged from 5 to 10% of the intake. This reduction reveals several metabolic differences between healthy individuals and patients with cancer and thus, reflects a higher consumption of ascorbate by cancerous cells. The Recommended Dietary Allowance (RDA) of AA of 200 mg per day is the required dose to saturate the body and thus, higher doses are considered to be unnecessary. A series of pharmacokinetic experiments conducted by the National Institute of Health showed that the plasma is saturated at around 70 μM after oral AA administration. Furthermore, it was shown that higher AA plasma concentrations are achieved by intravenous administration rather than by oral administration [13]. As a matter of fact, oral AA intake at the maximum tolerated dose (18 g/day), despite being given in high and frequent doses, achieves a maximum plasma concentration of 220 mol/L, whereas, high dose intravenous AA administration increases it to 14,000 mol/L [14]. Padayatty reported that intravenous doses of AA are able to saturate the plasma concentration 30- to 70-fold higher [15]. In fact, a plasma concentration of 15,000 μM might be reached after intravenous AA administration [13]. The mechanism of high dose intravenous AA is distinct from the high dose oral administration of AA. This is due to the limited intestinal absorption capacity which is bypassed by intravenous administration. This finding potentially explains the negative results shown in trials conducted at the Mayo clinic where only oral AA was administered [16]. Fifteen grams of intravenous AA increases plasma ascorbate levels by one or two orders of magnitude [14]. In fact, an AA plasma concentration of 1000–5000 mol/L is selectively cytotoxic to tumour cells in vitro [15,17,18,19]. When present at such high doses in plasma, which is only reachable by intravenous routes, AA generates the cytotoxic reactive oxygen species (ROS) hydrogen peroxide (H_2_O_2_). In non-cancerous cells, this is metabolized to water and oxygen. However, since tumour cells lack catalase, they are left vulnerable to the cytotoxic effects of H_2_O_2_. Besides being unable to convert H_2_O_2_, tumour cells selectively absorb more AA when compared to normal cells through facilitated transport by glucose transporters (GLUT) [7]. A recent study suggested that increasing intracellular iron using iron sucrose, which is usually given to colon cancer patients to attenuate anaemia, enhances the cytotoxic effect of AA on colon cancer cells. In vitro studies have revealed that in a comparison of the incubation of colon cancer cells with either pharmacological AA alone or AA following iron sucrose, levels of labile iron significantly increase ascorbate-induced augmentation of clonogenic colon cancer cells killing. Moreover, adding a combination of iron chelators, deferoxamine and diethylenetriaminepentaacetic acid, significantly inhibits the toxicity of either AA alone or AA following iron sucrose [20]. Another study showed that the lipophilic stable AA derivative, 2-*O*-α-d-glucopyranosyl-6-*O*-(2-pentylheptanoyl)-l-ascorbic acid (6-bOcta-AA-2G) is a better potent alternative drug to intravenous high-dose AA, especially for patients with renal failure. In vivo experiments in colon-26 tumour-bearing mice showed that the intravenous administration of 6-bOcta-AA-2G inhibited tumour growth more than AA [21].

### 3.3. Mechanism of Action of Ascorbic Acid in Cancer in General

The role of AA is likely to involve several functions. It induces pro-oxidant effects and inhibits energy metabolism, mainly mediated by H_2_O_2_ [22]. It acts as an electron donor and as a reducing agent which allows it to have a cytotoxic effect [23,24]. Furthermore, the pharmacologic doses of AA decrease the proliferation of RKO and SW480 colon cancer cells and induce their apoptosis and necrosis. This is mediated by the release of ROS that decreases the expression of the specificity protein (Sp) transcription factors Sp1, Sp3 and Sp4 and Sp-regulated genes which are involved in cancer cell proliferation (hepatocyte growth factor receptor (c-Met), epidermal growth factor receptor and cyclin D1), survival (survivin and bcl-2) and angiogenesis (vascular endothelial growth factor (VEGF) and its receptors (VEGFR1 and VEGFR2)) [25]. Another mechanism for inducing apoptosis by AA in colon cancer cells (HCT-8) is through an increase in the calcium influx in the endoplasmic reticulum, the translocation of Bad to the mitochondria from the cytosol after dissociation from 14-3-3β and an increase in the expression of Bax [26]. AA also serves as a cofactor for a large family of enzymes containing iron which are required for the regulation of HIF factors (required for tumour angiogenesis, treatment evasion and metastasis). Studies have shown that increasing intracellular ascorbate in vitro and vivo decreases HIF-1-activated reduced tumour growth and hypoxia [27].

In a study performed using xenograft models of different cancer cells to evaluate the cellular basis of the AA mechanism in cancer cell metabolism, Uetaki et al. found that the high-dose AA effect is mediated by H_2_O_2_ and depletes nicotinamide adenine dinucleotide (NAD), blocks the energy flux in glycolysis and the tricarboxylic acid cycle (TCA) cycle and consequently inhibits ATP production by increasing the upstream metabolites of glycolysis, the pentose phosphate pathway (PPP) and the partial TCA cycle (citrate and cis-aconitate) and decreases the downstream metabolites of glycolysis and TCA cycle metabolites with the exceptions of citrate and cis-aconitate and subsequently, decreases the ATP concentrations and adenylate energy charges [28]. In another study, the effect of AA was evaluated in three colon cancer cell lines of different genetic profiles. Even though AA was found to have anti-proliferative, cytotoxic and genotoxic effects on the three cell lines, the mechanism of cell death by AA differed either by increasing the level of oxidative stress or by utilizing a ROS-independent mechanism, as observed in LS1034 cells. AA cytotoxicity is influenced by the increased expression of AA transporters that allows the entrance of AA into cells, where it releases the electrons necessary for superoxide radical and H_2_O_2_ generation. It is also regulated by the mitochondria-dependent pathway mediated by a decrease in mitochondrial membrane potential (Ψm) and an increase in BAX/BCL-2 expression has been observed [29].

### 3.4. Mechanism of Action of Ascorbic Acid in Glycolysis, Particularly in CRC with Mutated BRAF/KRAS

At the cellular level, vitamin C has two forms: AA and its oxidized form, dehydroascorbate (DHA). Vitamin C in its AA form is transported into cells via sodium-dependent transporters 1 and 2 (SVCT1 and SVCT2), while its DHA form passes through facilitated diffusion via glucose transporters (GLUTs) that are upregulated in cancer cells due to the increased metabolic need for glucose [30].

The expression of the different SVCT transport proteins is tissue and cell type specific and is controlled by the transcriptional regulation of *SLC23* genes and post-translational regulation of the transporters [4]. Both SVCT1 and 2 play major roles in AA transportation where they co-transport sodium and ascorbate in a ratio of 2:1 down an electrochemical sodium gradient which is maintained by K/Na+ exchange mechanisms. They are capable of accumulating ascorbate against a concentration gradient from micromolar concentrations outside, to millimolar levels inside, the cells.

*KRAS* and *BRAF* mutations lead to activation via phosphorylation of the downstream Mitogen-Activated Protein Kinases/Extracellular Signal-Regulated Kinases (MAPK/ERK) pathway which, in turn, leads to the activation of transcription factors and gene expression, promoting cell proliferation and survival [31]. One of such genes that could be upregulated in these mutations is *GLUT-1*, which encodes the glucose transporter-1 protein. This allows such cells to have enhanced glucose uptake and glycolytic metabolism even at low glucose conditions which is something wild type CRC cells are unable to accomplish, thus glucose deficiency drives the acquisition of the *KRAS* pathway mutation [12]. Briefly, mutant *KRAS* is indeed involved in the upregulation of the Warburg effect in CRC cells.

**Figure 1:**
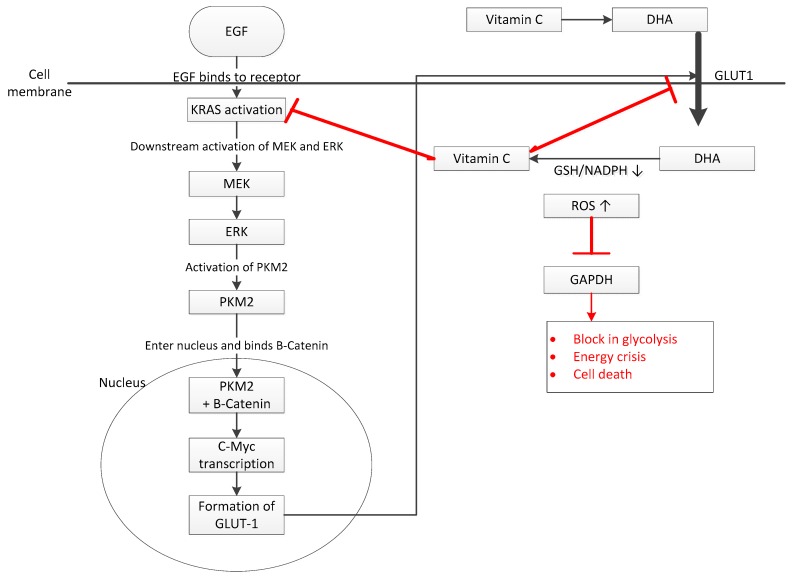
Effect of ascorbic acid (AA) on *KRAS* mutant colon cells. Upon entry into the cell, dehydroascorbate (DHA) is reduced to AA which has two effects: (**1**) RAS detachment from the cell membrane which inhibits the downstream phosphorylation cascade (MEK/ERK/PKM2) which, in turn, inhibits *GLUT-1* expression; and (**2**) glutathione depletion during the reduction process leads to accumulation of reactive oxygen species (ROS), which leads to the inactivation of GAPDH. These two effects inhibit glycolysis in cells that are highly dependent on energy, leading to an energy crisis and cell death. The effect of vitamin C is denoted in red.

Preclinical studies have shown that AA has a selective effect on CRC cells with *KRAS* or *BRAF* mutations, which are known to be mostly refractory to approved targeted therapies. Interestingly, high levels of AA selectively kill CRC cells harbouring these mutations and also impair their growth in mice [20]. Moreover, Hutton et al. demonstrated that cells carrying these mutations undergo metabolic reprogramming, leading to increased production of glycolytic proteins and glutamine proteins compared to wild type cells [32]. Studies on cell lines have demonstrated that upon entry into the cell, AA causes RAS to detach from the cell membrane, thereby inhibiting the entire downstream phosphorylation cascade (MEK/ERK/PKM2). This, in turn, inhibits several transcription processes, including those involved in *GLUT-1* expression and PKM2-PTB, an important regulator of the Warburg effect, leading to the inhibition of glycolysis, decreased ATP levels and effectively killing the cells (Figure 1) [33].

Experiments performed by Yun et al., on the other hand, determined a different mechanism of AA cytotoxicity via Glyceraldehyde-3-Phosphate Dehydrogenase (GAPDH) targeting. Increased *GLUT-1* transporter expression in mutated CRC cells leads to the accumulation of the oxidized form of AA, DHA, which, in turn, has to be reduced back to AA. This process depletes glutathione effectively, leading to the inability to clear up ROS which, in turn, targets an active cysteine site on GAPDH, leading to glutathionylation of this site and therefore, inactivation of GAPDH, an enzyme involved in glycolysis. The DNA damage induced by ROS activates Poly (ADP-ribose) polymerase, which, in turn, decreases NAD+ levels that further perpetuate GAPDH inhibition. These two effects inhibit glycolysis in cells that are highly dependent on energy, leading to an energy crisis and cell death [34].

Besides the cytotoxic effect of AA on tumour cells, AA was also found to beneficial in overcoming chemoresistance to cetuximab in mutated CRC cells. These cells express a high level of SVCT-2 protein which, in turn, allows for more AA uptake. AA, in combination with cetuximab, induces caspase-dependent apoptotic cell death as well as necrotic cell death. This combination was shown to inhibit the RAF and MAPK pathways and inhibit tumour growth as well. These effects were not demonstrated in vitamin C alone and in SVCT-2 negative cells. Hence, the therapeutic effect of vitamin C in combination with cetuximab depends entirely on the SVCT-2 levels of mutated CRC cells [35].

### 3.5. Previous Studies on Intravenous Ascorbic Acid Administration in Cancer in General

Several decades ago, intravenous AA administration was first introduced as a therapy for cancer by the Nobel Prize-winning Linus Pauling. In his study, longer survival was seen in advanced cancer patients treated with AA given orally and intravenously [36]. However, this finding was controversial with subsequent double-blinded, placebo-controlled studies from the Mayo Clinic using oral AA showing no advantage over placebo therapy in cancer patients [37]. Despite the controversy, AA continued to be considered as a complementary therapy in cancer [38]. Further studies showed that AA given intravenously in a dose greater than 0.5 grams bypasses the usual tight control of concentrations with oral doses. Therefore, peak plasma concentration is only reached by the intravenous route [39]. In a survey published in 2010 aiming to determine the reason for the use of intravenous AA administration, around 19% reported administering it as a treatment for cancer patients [40]. Ma and al. studied the role of AA use in combination with chemotherapy in patients with ovarian cancer. In preclinical models, the results were consistent with an anti-cancer effect and synergy with chemotherapy regimens. In human trials, results provided evidence of significant reduction in chemotherapy-induced adverse effects [41]. In another study, alternating intravenous and oral AA given to advanced cancer patients, showed that patients reported higher functional scores in terms of physical and cognitive functions and they reported lower symptom scores for nausea, vomiting and pain [42]. The results of the assessment of the quality of life questionnaire (QOL) developed by the European Organization of Research and Treatment of Cancer showed that intravenous AA can safely improve the quality of life of patients. It showed significant increase in physical, emotional and social functioning 3 weeks after the initiation of intravenous AA. It showed also a significant relief on the symptom scale, in terms of fatigue, pain, insomnia and constipation. Moreover, the reported adverse effects were mild and none of the patients stopped intravenous AA treatment due to the occurrence of adverse events [43]. In a cohort of patients with breast cancer taking intravenous AA throughout their chemotherapy regimen, significant decreases in fatigue, dizziness and loss of appetite were reported [44]. The safety profile of adding intravenous AA to standard treatment showed no increase in toxicity. It is contraindicated in patients with G6PD (glucose-6-phosphate dehydrogenase) deficiency due to the risk of haemolysis; yet, it is well tolerated in almost all patients [45]. It was reported that the ECOG scale (Eastern Cooperative Oncology Group performance status) was significantly better in a group of patients receiving intravenous AA jointly with standard chemotherapy compared to a placebo group. The tolerability was denoted to be excellent [46]. A number of case reports have been published that suggest potential clinical benefits from intravenous AA therapy. A case report of a 68-year-old newly diagnosed metastatic pancreatic cancer patient who refused to receive the conventional chemotherapy regimen and was treated with AA showed no disease progression at his 6-month follow up after surgery [47]. Another example is a case report of a 42-year-old man who was newly diagnosed with widely disseminated reticulum cell sarcoma. There was a delay in this patient receiving his conventional treatment and he was maintained on high dose intravenous AA as a “holding operation. Surprisingly, on the 10th day, the patient had symptoms improved and on the 22nd day, his chest X-ray was almost clear [48]. Seven months later, after a reduction in ascorbic acid intravenous dose, it is noteworthy that the disease was reactivated; therefore, a re-initiation of high-dose AA was used to re-induce a second complete remission [49]. When ascorbic acid was administered jointly with the conventional therapy, patients with multiple myeloma reported mostly mild to moderate adverse events (assessed as per the National Cancer Institute Common Toxicity Criteria) [50]. In a phase I trial conducted on stage IV pancreatic cancer in which fourteen patients were recruited, eight cycles of intravenous AA were administered in a dose escalation design along with concomitant standard treatment of gemcitabine and erlotinib. The results showed minimal adverse events overall, such as mild light-headedness or nausea [39]. Breast cancer patients given intravenous AA concomitantly with standard treatment benefited from a reduction in nausea, improved appetite and less fatigue. Patients in the study group that received intravenous AA reported symptoms, with the overall intensity score being nearly twice as low compared to the control group. They reported less therapy-induced complaints, as well as less depression and a reduction in sleep disorders [51]. Minimal adverse effects and toxicity were reported when AA was administered at escalating doses. In a phase I dose-escalating trial, the administration of ascorbic acid 1.5 g/kg three times weekly was reported to be essentially free of risk. A case report on advanced ovarian cancer showed that adding intravenous AA adjunctively to first-line chemotherapy, might improve the efficacy of chemotherapy [19]. There appears to be a growing number of studies showing improvement in the quality of life and an increase in the survival times of terminal cancer patients [52]. An association was found between a deficiency in plasma AA and an increased tumour size in endometrial cancer and thus, ascorbate accumulation decreased with higher tumour grades [53]. Recent studies showed that a low plasma AA concentration is significantly associated with high levels of the inflammatory marker C-reactive protein (CRP) from one side and positively correlated with survival rates from the other side. As a matter of fact, AA deficiency is common in 30% of patients with advanced cancer [54]. In hormone-refractory prostate cancer, a daily intraperitoneal administration of AA for 30 days led to a significant reduction in tumour size and a reduction in pulmonary and lymphatic metastasis [55]. Two case reports on metastatic renal cell carcinoma described in the late 1990s showed the resolution of metastatic lesions after the initiation of intravenous AA as a sole therapy; one of the patients remained cancer-free for 14 years and died from congestive heart failure [56]. Supplementation of AA might benefit the quality of life of cancer patients [57]. A recent case report discussed a newly-diagnosed stage IV poorly-differentiated pancreatic ductal adenocarcinoma. This 68-year-old male patient electively chose to be treated with escalating doses of intravenous AA as his sole treatment as an exclusive regimen, declining the conventional standard of care. The doses of AA ranged from 75 to 125 g per infusion and were administered 2–3 times per week (after being screened for G6PD deficiency or abnormal renal function). The results were outstanding; the patient achieved objective regression of his disease and he survived nearly 4 years after diagnosis. He died from a bowel perforation event leading to sepsis and organ failure [58]. Decreased levels of the inflammatory marker CRP are seen in patients under intravenous AA therapy. Cancer patients who are deficient in AA tend to have lower plasma concentrations after infusion; in fact, high tumour burdens measured by CRP and other markers tend to decrease AA plasma levels. Levels vary depending on the tumour degree of inflammation. Therefore, evidence suggests that intravenous AA might serve as a safe adjunctive therapy and it might have a role in modulating the inflammation, which, in turn, may improve patient outcomes [14]. A phase I clinical trial was conducted in the USA in which biopsy-proven metastatic pancreatic adenocarcinoma patients were given AA intravenously in addition to gemcitabine. The results revealed that the average survival time was 13 ± 2 months, compared to survival times of 5.65 months for patients in similar cases [45,59]. In a study in which different types of cancer patients whose treating oncologist decided that the standard-of-care offered less than a 33% likelihood of disease response received intravenous AA with concomitant chemotherapy, the results showed increased energy and functional improvement; no intravenous AA-related toxicity was reported [60]. High dose intravenous AA therapy affects pro-inflammatory markers. There is a significant reduction in CRP and in pro-inflammatory cytokines (such as IL-1α, IL-2, IL-8, TNF-α) when intravenous AA s administered after standard treatments by conventional methods in patients with different cancer types (prostate cancer, breast cancer, bladder cancer, pancreatic cancer, lung cancer, thyroid cancer, skin cancer and B-cell lymphoma). A study conducted by Mikirova and al. supported the hypothesis that high-dose intravenous AA therapy reduces inflammation in malignant diseases [61]. In a pilot clinical study, twenty-four end-stage cancer patients received continuous intravenous AA infusion over eight weeks. These patients were AA deficient prior to therapy. The results demonstrated that plasma AA increased and patients did not report any renal distress, provided no history of kidney problems pre-existed before the initiation of the treatment. The most frequently reported minor adverse events were nausea, oedema and dry mouth and skin. Therefore, this study supports the safety of intravenous AA therapy provided the patient does not have a history of kidney stone formation [62]. Safety data from a phase I study on advanced pancreatic cancer showed that in the absence of renal failure, the overall reported adverse events are not greater than disease-related or chemotherapy-related adverse events. This was a study in which patients were divided into three cohorts; the first cohort received 50 grams of intravenous AA, which escalated to 75 grams in the second cohort until reaching the third one with 100 grams. In addition to AA, standard chemotherapy was administered concomitantly. The results showed that there were no ascorbic acid toxicity-related symptoms [63].

A summary of previous studies on the use of intravenous AA in cancer is listed in Table 1.

### 3.6. Previous Studies on Intravenous Ascorbic Acid Administration in Colorectal Cancer

The level of plasma AA is lower in patients with colorectal cancer compared to healthy subjects [64]. In a randomized, double-blinded study conducted between November 2013 and October 2014, Jeon et al. assessed the effect of intravenous AA on post laparoscopic colectomy pain in colon cancer patients who were given AA intravenously after the induction of anaesthesia. The results showed that a high intravenous AA dose decreases pain within 24 h post-op. Compared to the placebo group, morphine consumption in the early post-operative phase seemed to be reduced as well. The analgesic effect of AA might be related to its ability as an antioxidant to inhibit the creation of ROS, which, in turn, is a known factor in neuropathic pain [65] (Table 2). A case report in 2004 described a stage IV poorly-differentiated colon adenocarcinoma with stomach wall and liver metastatic lesions. The patient was determined to have very poor prognosis. He started intravenous administration of 15 grams of AA per hour combined with second line adjuvant chemotherapy. The intravenous AA administration doses were gradually increased during bi-weekly infusions until they reached 100 grams twice weekly. On his one-year follow up, the patient was found to be disease free. In terms of side effects, only chemotherapy-related effects were reported during a hiatus from intravenous AA that disappeared upon reinstatement of intravenous AA. None of the common side effects of leucopoenia, thrombocytopenia, or anaemia were ever experienced [56]. Preclinical studies have shown that mutant *KRAS* and *BRAF* cells, which are known to be refractory to approved targeted therapies, are selectively killed by high levels of ascorbic acid. High dose ascorbic acid impaired tumour growth in mutant cells [34]. Previous phase I–II clinical trials have found that high dose intravenous ascorbate acid (1.5 g/kg) is well tolerated in cancer patients [60].

## 4. Conclusions

Ascorbic acid inhibits glycolysis either by blocking one of its essential enzymes or by shutting down the pathway that allows for enhanced glucose absorption which is dependent on the *KRAS* mutational status. Ascorbic acid was found to be beneficial for the treatment of cancer in small studies, only when administered intravenously and in high and frequent doses. When given in combination with standard therapy and in the absence of renal failure, the overall reported adverse events were not greater than therapy-induced complaints. There was no increase in toxicity; yet, the results were promising and decreased levels of the inflammatory marker CRP were shown, as well as an improvement in the efficacy of chemotherapy and thus, an increase in survival times. Moreover, patients reported an improvement in quality of life and increased energy.

A limited number of clinical studies are available on the effect of intravenous ascorbic acid in colon cancer patients. We have outlined the preclinical rationale for activity in this disease. The clinical evidence remains limited, and thus, the need to conduct further clinical trials on a larger scale is high.

## Figures and Tables

**Table 1:** Clinical studies of ascorbic acid (AA) in cancer in general.

Condition	Type of Study	Study Sample	Concomitant Therapy	Finding	Reference
Stage IV pancreatic cancer	Phase I open-label, dose-escalating trial	14	Gemcitabine/Erlotinib	- No increase in toxicity - Minimal adverse events (such as mild light-headedness or nausea)	[39]
Stage IV pancreatic cancer	Case report	1	None	Objective regression of the disease	[58]
Stage IV pancreatic cancer	Phase I clinical trial	9	Concurrent Gemcitabine	- Mean survival 13+ months - Time to progression 26 ± 7 weeks	[59]
Stages II–III breast cancer	Epidemiological, retrospective and observational study	125	Standard tumour therapy	- No side-effects of intravenous AA - Reduction in quality of life-related side-effects	[51]
Stage IV renal cell carcinoma	Case reports	2	None	Resolution of metastatic lesions	[56]
Terminal cancer	Nonrandomized clinical trials	99	None	- Increase in survival times - Improved quality of life	[52]
Different types of cancer	Phase I–II clinical trial	14	Standard tumour therapy	- Increased energy and functional improvement - No intravenous AA-related toxicity	[60]
Different types of cancer	Observational study	45	None	Reduction in pro-inflammatory cytokines	[61]

**Table 2:** Clinical studies of AA in colorectal cancer.

Condition	Aim of the Study	Finding	Reference
Colon cancer (Duke B–C stages)	- Compare the oxidation and antioxidant potential of patients with CRC - Determine plasma vitamin E and C levels in CRC	Level of plasma vitamin C is lower in patients with CRC when compared to healthy subjects.	[64]
Resectable colon cancer	Effect of intravenous AA on post laparoscopic colectomy pain	High dose intravenous AA decreases pain within 24 h post-op.	[65]
Colon cancer (stage IV) and other cancer types	Case report on the effects of intravenous AA given alone or concomitantly with chemotherapy	- Disease free after 1 year - Only chemotherapy-related side effects were reported during a hiatus from intravenous AA that disappeared upon reinstatement of intravenous AA.	[56]

## Abbreviations

6-bOcta-AA-2G	2-*O*-α-d-glucopyranosyl-6-*O*-(2-pentylheptanoyl)-l-ascorbic acid
AA	Ascorbic acid
CRC	Colorectal cancer
CRP	C-reactive protein
DHA	Dehydroascorbate
EGFR	Epidermal growth factor receptor
GAPDH	Glyceraldehyde-3-Phosphate Dehydrogenase
GLUT	Glucose transporters
G6PD	Glucose-6-phosphate dehydrogenase
H2O2	Hydrogen peroxide
HIF	Hypoxia-inducible transcription
HK-2	Hexokinase-2
IL	Interleukins
MAPK/ERK	Mitogen-Activated Protein Kinases/Extracellular Signal-Regulated Kinases
MEK/ERK/PKM2	Phosphorylation cascade
NAD	Nicotinamide adenine dinucleotide
PDK1	Pyruvate dehydrogenase kinase isozyme 1
PPP	Pentose phosphate pathway
ROS	Reactive oxygen species
Sp	Specificity protein
STAT	Signal transducer and activator of transcription
SVCT1	Sodium-dependent vitamin C transporters 1
SVCT2	Sodium-dependent vitamin C transporters 2
TCA	Tricarboxylic acid cycle
VEGF	Vascular endothelial growth factor
Wnt	Wingless-related integration site
Ψm	Mitochondrial membrane potential
